# Genome-wide association study of myocarditis and pericarditis following COVID-19 vaccination

**DOI:** 10.1038/s41541-025-01139-4

**Published:** 2025-05-08

**Authors:** Marco Cavalli, Niclas Eriksson, Tomasz Baron, Ahmet Yalcinkaya, Nils Landegren, Petter Brodin, Pär Hallberg, Mia Wadelius

**Affiliations:** 1 Department of Medical Sciences, Clinical Pharmacogenomics, Science for Life Laboratory, Uppsala University, Uppsala, Sweden; 2 Department of Immunology, Genetics and Pathology, Science for Life Laboratory, Uppsala University, Uppsala, Sweden; 3 Uppsala Clinical Research (UCR) center, Uppsala, Sweden; 4 Department of Medical Sciences, Cardiology, Uppsala Clinical Research Centre, Uppsala University, Uppsala, Sweden; 5 Science for Life Laboratory, Department of Medical Biochemistry and Microbiology, Uppsala University, Uppsala, Sweden; 6 Department of Medical Biochemistry, Hacettepe University Hospital, Ankara, Turkey; 7 Center for Molecular Medicine, Department of Medicine (Solna), Karolinska Institutet, Stockholm, Sweden; 8 Unit for Clinical Pediatrics, Department of Women’s and Children’s Health, Karolinska Institutet, Stockholm, Sweden; 9 Department of Immunology & Inflammation, Imperial College London, London, United Kingdom

**Keywords:** Genetics research, Inflammasome

## Abstract

This genome-wide association study (GWAS) explores the genetic components of severe adverse events following COVID-19 vaccination, with focus on myocarditis and pericarditis. Three SNPs (rs536572545, rs146289966 and rs142297026) near the *SCAF11* gene were linked to pericarditis, while rs570375365 in the *LRRC4C* gene was associated with myocarditis. These findings suggest that genetic variants may influence inflammation pathways, providing a basis for further investigation into the immunological responses triggered by vaccines.

The first case of severe acute respiratory syndrome due to coronavirus 2 (SARS-CoV-2) infection was identified in Wuhan, China in December 2019. The virus spread globally, and on March 11, 2020, the World Health Organization (WHO) declared the coronavirus disease-2019 (COVID-19) outbreak a global pandemic^1^. In response, several vaccines were rapidly developed, including inactivated viruses (Covilo, CoronaVac, and Covaxin), protein subunits (Nuvaxovid/Covovax), viral vectors (Janssen/Jcovden and Vaxzevria/Covishield), and for the first time, mRNA vaccines (Pfizer BNT162b2/Comirnaty and Moderna mRNA-1273/Spikevax).

In Sweden, four COVID-19 vaccines were available: Comirnaty, Spikevax, Nuvaxovid, and Vaxzevria. The Swedish Health Agency ceased recommending Vaxzevria in September 2021 and discontinued Spikevax for individuals under 30 years in October 2021 due to the increased risk of myocarditis and pericarditis. As of May 2024, 105,919 suspected adverse events following immunization (AEFIs) related to COVID-19 vaccines have been reported to the Swedish Medical Products Agency (MPA), with ~76% linked to mRNA vaccines^2^.

Most AEFIs linked to COVID-19 vaccines are mild and self-limiting, such as fatigue, headache, myalgia, fever, chills, and joint pain, while moderately severe allergic reactions have also been reported. Severe AEFIs are exceedingly rare, but include several notable conditions such as myocarditis and pericarditis^3^, thrombocytopenia, thromboembolism^4^, neurological disorders^5^, and anaphylaxis. In particular, public concern has focused on myocarditis and pericarditis after mRNA vaccination that disproportionately developed among young males (aged 16 years and older) within 7 days of the second dose^6^. Myocarditis following vaccination could be associated with both innate and adaptive immune response to the SARS-CoV-2 Spike glycoprotein, but also with the direct impact of the mRNA administered by the vaccine^7^. The higher incidence among young men suggests that sex hormones and genetic differences may play a role^6^. However, the pathogenic mechanism remains poorly understood, making it crucial to investigate underlying patient characteristics to develop preventive and therapeutic methods.

SWEDEGENE (www.swedegene.se) is a Swedish national biobank established in 2009 to study genetic and clinical factors contributing to adverse drug reactions (ADRs). It has collected clinical data and blood samples from about 5000 cases of various ADRs. In April 2021, SWEDEGENE began recruiting patients diagnosed with AEFIs after receiving Comirnaty, Spikevax, Vaxzevria, and Nuvaxovid vaccines, creating the SWEDEGENE-COVID19 cohort. Sampling included blood, plasma, and peripheral blood mononuclear cells (PBMCs). As of June 2024, 890 cases have been recruited.

This study aimed to explore genetic and immunological factors underlying AEFIs using a genome-wide association study (GWAS) on a sub-cohort with myocarditis, pericarditis, and perimyocarditis following COVID-19 vaccination (SWEDEGENE-COVID19-MYOPERIC). The diagnoses of the cases in this study were made at the discretion of the treating physicians based on clinical presentation and available cardiac testing, rather than a predefined systematic approach (see Methods/Hospital care). Demographics, clinical characteristics, and vaccination parameters for this sub-cohort are presented in Table 1. The GWAS analyses were performed independently of vaccine type, for only mRNA vaccines (Comirnaty+Spikevax), and for each vaccine independently.

**Table 1:** Demographics, clinical characteristics and vaccination parameters for the SWEDEGENE-COVID19-MYOPERIC cohort

Demographics	All cases	Myocarditis	Pericarditis	Perimyocarditis
Male, *n*	44	24	11	9
Female, *n*	22	11	7	4
Ethnicity, Swedish, *n* (% of total)	57 (86.3)	30 (85.7)	17 (94.4)	10 (83.3)
Age in years, mean ± SD (range)	41.2 ± 19.2 (18–87)	39.6 ± 18.1 (18–77)	50.4 ± 19 (18–87)	33.1 ± 17.2 (18–72)
≤25 years, *n* (% males)	19 (84.2)	10 (80)	2 (100)	7 (85.7)
26-50 years, *n* (% males)	25 (68)	16 (62.5)	6 (83.3)	3 (66.6)
>50 years, *n* (% males)	22 (50)	9 (66.6)	10 (40)	3 (66.6)
**Clinical characteristics at the time of the AEFI**				
Weight in kg, mean ± SD (range)	76.3 ± 15.6 (52–120)^a^	77.6 ± 14.9 (54–115)	72.4 ± 13.7 (53–105)	77.9 ± 18.9 (52–120)^a^
Height in cm, mean ± SD (range)	175.7 ± 10.7 (155–201)	176.9 ± 10 (155–201)	173.2 ± 11.1 (158–197)	176.6 ± 11.3 (159–200)
BMI, kg/m^2^, mean ± SD (range)	24.6 ± 3.9 (19.3–40.1)^a^	24.6 ± 3.2 (19.4–33.9)	24 ± 3.5 (19.7–32.7)	25.4 ± 5.6 (19.3–40.1)^a^
Smoker, *n* (% of total)	1 (1.5)	0 (0)	1 (5.5)	0 (0)
Alcohol standard glasses/week, mean ± SD (range)	3.5 ± 4.1(0–19)^b^	3.6 ± 4 (0–15)^a^	2.3 ± 2.3 (0–9)	4.7 ± 5.7 (0–19)^a^
**Vaccination parameters**				
Time to onset in days, mean ± SD (range)	8.8 ± 9.2 (0–37)^a^	8.2 ± 8.9 (0–37)^a^	12.1 ± 10 (1–36)	5.5 ± 7.2 (0–27)
Type of vaccine and dose associated with AEFI				
Comirnaty (dose 1)	12	2	5	5
Comirnaty (dose 2)	21	11	6	4
Comirnaty (dose ≥ 3)	8	5	3	0
Spikevax (dose 1)	9	6	1	2
Spikevax (dose 2)	11	7	3	1
Spikevax (dose ≥ 3)	3	3	0	0
Vaxzevria (dose 1)	2	1	0	1
Vaxzevria (dose 2)	0	0	0	0
Vaxzevria (dose ≥ 3)	0	0	0	0
**Hospitalization following AEFI**				
Length of hospitalization in days, mean ± SD (range)	4.3 ± 4.8 (0–31)^c^	3.6 ± 2.4 (0–10)^d^	6.8 ± 8 (0–31)^e^	2.8 ± 1.4 (0–5)
*Treatment*				
Standard cardiac exams & monitoring^f^, *n* (% of total)	34 (51.5)	20 (57.2)	6 (33.3)	8 (61.5)
Additional specific treatments^g^, *n* (% of total)	29 (44)	13 (37.1)	11 (61.1)	5 (38.5)
Missing info, *n* (% of total)	3 (4.5)	2 (5.7)	1 (5.6)	0 (0)
*Outcome*				
Fully recovered, *n* (% of total)	27 (41)	15 (42.8)	4 (22.2)	8 (61.5)
Time elapsed in months, mean ± SD (range)	2.7 ± 1.84 (1–8.5)	2 ± 0.8 (1–7)	3 ± 0.8 (1–4)	3 ± 2.2 (1–8.5)
Improved, not recovered, *n* (% of total)	21 (31.8)	7 (20)	10 (55.5)	4 (30.8)
Time elapsed in months, mean ± SD (range)	2.5 ± 1.67 (0.5–8)	3.3 ± 0.5 (2–4)	1.2 ± 0.2 (0.5–4)	3.8 ± 2.7 (1–8)
Not recovered, *n* (% of total)	3 (4.5)	3 (8.5)	0 (0)	0 (0)
Time elapsed in months, mean ± SD (range)	2.3 ± 0.47 (2–3)	2.3 ± 0.47 (2–3)	0	0
Missing info, *n* (% of total)	15 (22.7)	10 (28.6)	4 (22.2)	1 (7.7)

^a^Missing data for one patient.

^b^Missing data for two patients.

^c^Missing data for five patients.

^d^Missing data for three patients.

^e^Missing data for two patients.

^f^Coronary arteries angiography, CT-scans, EKG, Echo, cardiac MRI, cardiac telemetry.

^g^Defibrillator, ICU stay, specific drugs treatment.

In the overall analysis (*n* = 66), we observed a significant association with three SNPs located within or close to the *SCAF11* gene (Supplementary Fig. 1 and Supplementary Data 4): rs536572545 (intronic), rs146289966 (upstream), and rs142297026 (downstream). The odds ratio (OR) for rs536572545 and rs146289966 was 110 [95% confidence interval (CI) 27.7, 439], *p* = 1.11 × 10^–8^, while the OR for rs142297026 was 82 [95% CI 22.3, 305], *p* = 3.28 × 10^–8^. The results were similar when restricting the analysis to Comirnaty+Spikevax cases (*n* = 64, Supplementary Fig. 2 and Supplementary Data 5) where rs536572545 and rs146289966 had an OR of 115 [95% CI 28.9, 458], p = 9.33×10^–9^, while rs142297026 had an OR of 86 [95% CI 23.3, 320], *p* = 2.7 × 10^–8^. No SNP passed the significance threshold when stratified by individual vaccines (data not shown).

rs536572545, is located in the first intron of *SCAF11* (SR-Related CTD Associated Factor 11), and is in linkage disequilibrium (LD) with rs146289966 (R^2^ = 0.75) which overlaps a transcription factor (TF) motif for ZNF701. rs142297026 is in high LD (R^2^ > 0.8) with three SNPs located in introns of *ARID2* (AT-rich interactive domain-containing protein 2): rs548055420 (intron 3), rs147643073 (intron 4) and rs150021835 (intron 16). Additionally, rs147643073 overlaps TF motifs for IRF4, IRF1, IRF8, IRF7, IRF5, IRF9, STAT2 and PRDM4, while the *ARID2* SNP rs150021835 overlaps a TF motif for ZNF382. Both *SCAF11* and *ARID2* are expressed ubiquitously.

*SCAF11* is involved in spliceosomal complex assembly and is considered a pyroptosis-related gene^8^, associated with highly inflammatory programmed cell death. Pyroptosis, characterized by the release of pro-inflammatory cytokines and inflammasome activation, has been implicated in various diseases, including cardiovascular diseases and viral myocarditis^9^. A role for pyroptosis has also been shown in hyperinflammation in COVID-19^10^, and excessive or uncontrolled pyroptosis can contribute to tissue damage and inflammation. Anti-inflammatory treatments in the form of interleukin blockers (mainly against IL-1) and inflammasome inhibitors (e.g. colchicine) have proven therapeutic efficacy in patients with pericarditis^11,12^. This suggests that *SCAF11* is a target for further studies aiming to elucidate the pathogenesis of pericarditis attributed to COVID-19 vaccination. Less is known regarding the contribution of ARID2, a chromatin remodeler, to inflammation. Notably, *Arid2* knockout mouse models show enrichment of inflammatory pathways with upregulation of Toll-like receptors^13^ and a long noncoding RNA (*Arid2-IR)* has been linked to renal inflammation in mice^14^.

Next, considering all pericarditis (including perimyocarditis) cases (*n* = 31), significant associations were observed for the same three SNPs within or close to the *SCAF11* gene: rs536572545, rs146289966, rs142297026 (Fig. 1a and Supplementary Data 6). For rs536572545 and rs146289966, the OR was 201 [95% CI 45.9, 883], *p* = 1.91x10^–8^, while the OR for rs142297026 was 158 [95% CI 38.8, 645], *p* = 3.97x10^–8^. Again, the association appeared to be driven by Comirnaty+Spikevax cases (*n* = 30, Supplementary Fig. 3 and Supplementary Data 7) for which rs536572545 and rs146289966 had an OR of 213 [95% CI 48.7, 935], *p* = 1.47x10^–8^, and rs142297026 had an OR of 168 [95% CI 41.4, 688], *p* = 2.98x10^–8^. No SNP passed the significance threshold when the analysis was stratified into individual vaccines (data not shown).

**Fig. 1:**
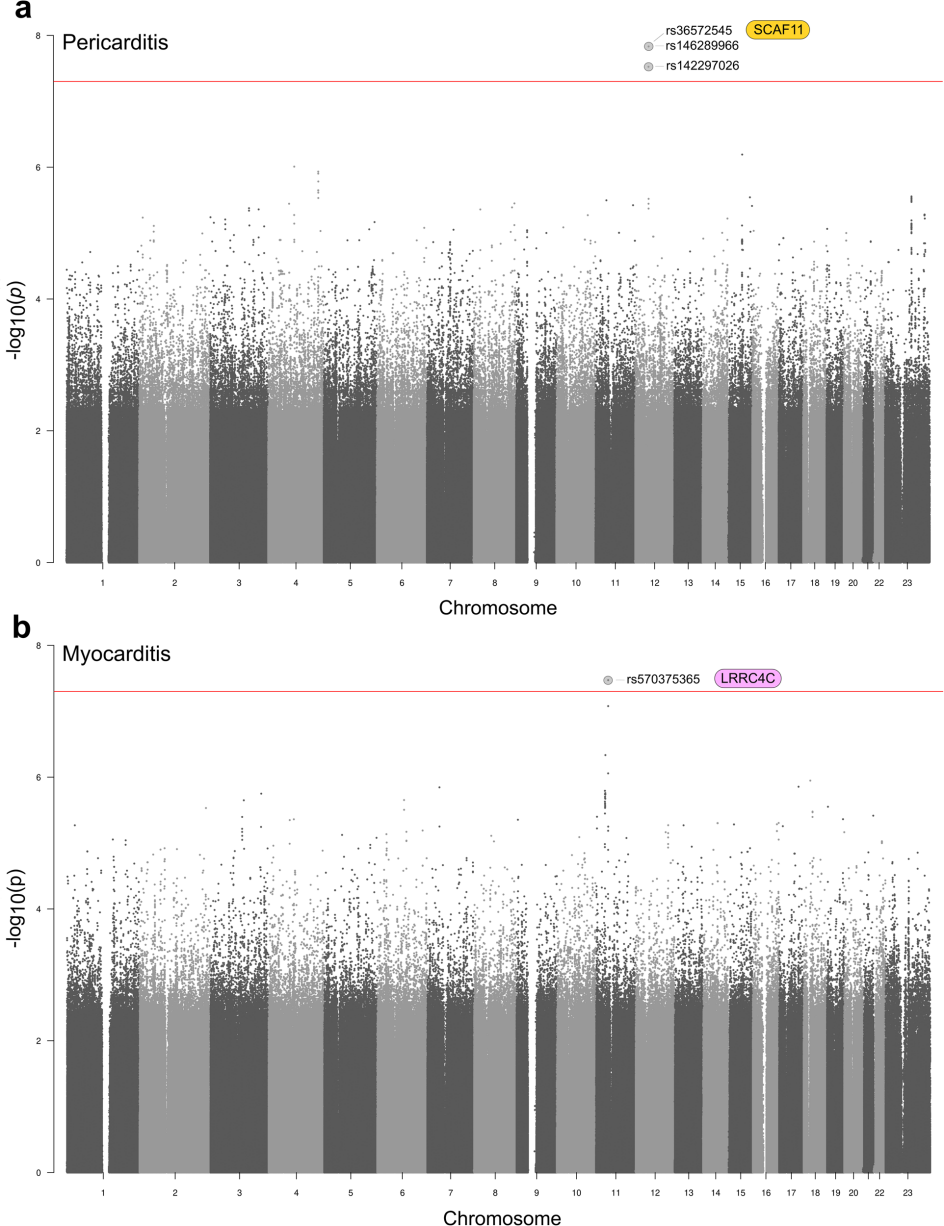
SAIGE GWAS for pericarditis and myocarditis cases. Manhattan plots for (**a**) 31 cases of pericarditis cases (including perimyocarditis) from all types of COVID-19 vaccines, and (**b**) 19 cases of myocarditis (including perimyocarditis) associated with Spikevax COVID-19 vaccine compared with 4891 population controls. The red line denotes the significance level (*p* < 5×10^–8^).

Finally, for myocarditis (including perimyocarditis), we observed a significant association at a genome-wide level only between Spikevax cases (*n* = 19) and the SNP rs570375365 in the *LRRC4C* gene, OR 21.5 [95% CI 9.0, 51.0], *p* = 3.37x10^–8^ (Fig. 1b and Supplementary Data 8). rs570375365 is located in the first intron of *LRRC4C* and in proximity to a 27 kb region that contains 13 candidate cis regulatory elements (cCREs) annotated as enhancers and defined by DNase I hypersensitive sites (DHSs) signals in heart tissue samples in ENCODE3. It overlaps a TF motif for ZNF460.

*LRRC4C*, which encodes netrin-G1 ligand (NGL-1), is mainly expressed in the brain but also in the heart^15^. *LRRC4C* expression acts as a prognostic marker in cancer and is linked to immune-related signaling pathways^16^. Missense variants in *LRRC4C* have also been significantly associated with susceptibility^17^ and severe outcome of COVID-19^18^. The GWAS SNP we identified flags a genomic region in the first intron of *LRRC4C* with multiple annotated enhancers that should be further investigated experimentally to determine a possible contribution of *LRRC4C* to the inflammatory process in myocarditis cases.

The overall GWAS results highlight genes, genomic regions, and inflammatory pathways that may serve as starting points for further studies using proteomic and inflammation assays to understand the causes of myo-/pericarditis associated with COVID-19 vaccines. A focus will be on autoimmunity with autoantibody screenings between cases and healthy controls. We have previously shown that the recruited patients were negative for type I IFN autoantibodies, suggesting that myo-/pericarditis AEFIs to COVID-19 vaccines cannot be explained by autoantibody-mediated neutralization of IFN signaling^19^.

From a clinical point of view, identifying specific pathways or molecules (e.g., cytokines) associated with myo-/pericarditis could pinpoint new therapeutic targets for the treatment and/or prevention of these severe AEFIs. For example, if overexpression of certain cytokines is detected, cytokine inhibitors might be used in a similar fashion to how cytokine storms are managed during COVID-19 infections. The strategy outlined here can be adapted to various types of AEFI with the ultimate goal of optimizing the use of medicines and medical resources. This approach could also facilitate personalized decision-making in future immunization campaigns during pandemics.

The main limitation of this study is the number of cases, which reflects the rarity of the events in the relatively small Swedish population. Nonetheless, the characteristics of the recruited patients, such as a prevalence of young males who experienced a myo-/pericarditis approximately one week after the second vaccine dose are similar to those observed worldwide. In addition to the limited cohort size, proving causality is a critical challenge. While the WHO describes criteria to assess causality for potential AEFIs, the definition of ‘temporal proximity’ remains uncertain. The temporal window from vaccination to diagnosis of an AEFI is a dynamic range that is continuously updated as more cases are described. To overcome this potential confounder, we used the most stringent temporal windows for AEFIs that had been reported in the literature to maximize the likelihood of a causal link. Another notable limitation of our study is the reliance on clinical and imaging diagnoses of myocarditis without the uniform application of standardized criteria such as the ones from the American College of Cardiology (ACC)^20^, European Society of Cardiology (ESC)^21^, or Brighton Collaboration diagnostic criteria^22^. This approach may introduce variability in case identification and classification, potentially affecting the accuracy and generalizability of our findings.

In conclusion, this study which comprised patients who had experienced myo-/pericarditis after immunization with COVID-19 vaccines, has identified several notable genetic variants that may help generate hypotheses about pathogenic mechanisms underlying these serious AEFIs. The results need further validation and replication in a larger, independent cohort to confirm the association and clarify the effect size estimates.

## Methods

### Cohort description

#### Patient recruitment

Inclusion criteria were patients aged 18 years or older at the time of recruitment and having experienced myocarditis, pericarditis, or perimyocarditis as suspected AEFIs to COVID-19 vaccines. The exclusion criterion was inability to provide informed consent.

Cases were identified through the Swedish MPA’s national database of spontaneously reported ADRs from health care professionals. Clinical data were obtained from medical records and a standardized questionnaire. Blood, plasma and/or saliva samples were taken at a clinical laboratory of the patient’s choice, and sent for storage at Uppsala University Hospital (Uppsala Biobank), and/or at Karolinska Institutet (KI Biobank).

A letter was sent to physicians who reported a suspected case to the MPA. This letter explained the objectives of the project and asked the reporter whether the patient could be contacted. If there was no objection, the patient was sent a letter of invitation to participate in the study. A research assistant next phoned the patient to ask about participation in the study. Consenting participants were sent a study kit containing everything necessary to acquire blood samples, a copy of a questionnaire collecting information about ethnicity, medical history, lifestyle questions, and information about drug treatment, as well as an informed consent slip.

The questionnaire was completed through a phone interview with a research assistant. Patients who for any reason, were not willing or able to draw blood were offered the possibility of saliva sampling.

Copies of medical records relevant for the assessment of the AEFI were obtained for each case, including medical notes, drug treatments, X-ray data if available, and laboratory examinations. Once all relevant clinical data had been received and reviewed, they were entered into a study database.

### Causality assessment

To address the possible causal relationship between COVID-19 vaccination and the AEFI, we followed the AEFI causality assessment tool published by the WHO (2nd ed., 2019 update, https://www.who.int/publications/i/item/9789241516990). Briefly, for each suspected case of myocarditis, pericarditis, or perimyocarditis, we addressed five questions: (I) eligibility: *is the diagnosis valid?*; (II) order of incidence: *was the vaccine administered before the cardiac AEFI?*; (III) temporal proximity: *did the AEFI happen within a plausible time window after the vaccination?* In this study we used a window of up to 42 days based on literature reports; (IV) evidence for other causes: *are there other causes that work against a causal association (e.g. other diseases in the background)?*; and (V) published evidence of a causal association: *have other cases of the AEFI been reported?*

Based on these questions, each case was determined to be “definitely related”, “possibly related” or “unlikely related” to the vaccines. In this study, we used only cases assessed as “definitely related” and “possibly related”. Five cases were assessed as “unlikely related” based on unlikely temporal proximity (an average of 119 days) from the vaccination to the diagnosis and were excluded. The cases’ causality assessment was performed in a two-tier rating step, first by medical doctors at SWEDEGENE and then by an independent cardiologist consultant at Uppsala University Hospital who, in case of disagreement, made the final decision in the assessment. The two independent causality assessment were in agreement for all 66 cases recruited into the SWEDEGENE-COVID19-MYOPERIC cohort.

A total of 4891 unrelated individuals from the Swedish Twin Registry (Twingene) were used as population controls^23^. These were of predominantly Swedish origin and were born between 1911 and 1958.

### Data collection and variables

As of September 2023, the SWEDEGENE-COVID19-MYOPERIC cohort comprised 66 patients who had experienced myocarditis (*n* = 35), pericarditis (*n* = 18) or perimyocarditis (*n* = 13) following COVID-19 vaccination. A summary of the case characteristics by AEFI is presented in Table 1. Two thirds of the patients were males, while 84% were males in the youngest age group (18–25 years), which is in line with findings reported by the US Centers for Disease Control and Prevention (CDC)^24^. In the great majority of cases, an mRNA vaccine was the suspected vaccine. We observed a higher frequency of reported AEFIs after the second dose. The average time to onset after immunization was 8.8 days, which is close to the commonly reported window of about one week^6^.

### Hospital care

All recruited patients required hospitalization with stays ranging from a few hours to several days. Diagnostic validity has been shown to be high in Swedish hospitals, particularly >95% for myocarditis cases^25^.

An overview of the diagnostic investigations of the myo-/pericarditis cases is presented in Table 1 and Supplementary Data 1. It is important to note that the diagnosis of myocarditis in our study was based on clinical presentation and imaging findings, without systematic application of the latest ACC, ESC, or Brighton Collaboration diagnostic criteria. Briefly, all patients were subject to standard cardiac exams such as coronary artery angiography, computed tomography (CT) scans of the thorax, lungs, or heart, electrocardiography (ECG), echocardiography, and cardiac magnetic resonance imaging (MRI), and they were often monitored via cardiac telemetry or Holter monitors. In addition, laboratory analyses included cardiac troponins, N-terminal prohormone of brain natriuretic peptide (NT-proBNP), C-reactive protein (CRP), leukocytes, and other biomarkers of cardiac injury or inflammation. Twenty-nine patients (44% of the total) required specific treatments. These included beta-blockers (metoprolol, bisoprolol), ACE inhibitors (enalapril, ramipril), calcium channel blockers (felodipine), angiotensin II receptor antagonists (candesartan), and diuretics (furosemide, eplerenone, spironolactone). Other common treatments included anti-inflammatory drugs (colchicine), anticoagulants or antiplatelet drugs (apixaban, acetylsalicylic acid), and corticosteroids (betamethasone, prednisolone). Three cases with myocarditis required advanced life support in the intensive care unit (including defibrillation).

The outcome of the treatments was inferred from the medical records, when available, and based on follow-up hospital visits. Across the three AEFI subgroups, we observed an average recovery time of less than 3 months.

### Medical history and medications in the prior three months

Medical histories (including any drug treatments) in the three months before COVID-19 vaccination were obtained by questionnaires and medical records. As expected, we observed that reported comorbidities were positively correlated with patient age. Notably, cardiac, gastrointestinal, autoimmune, respiratory, and thyroid diseases, as well as high blood pressure, were the most common (Supplementary Data 2a and b).

Thirty percent of the recruited patients were not taking medications in the three months prior to AEFI onset. Over-the-counter drugs such as paracetamol and NSAIDs (e.g., ibuprofen, naproxen, aspirin) were mainly taken by younger patients. Other frequently reported drugs were respiratory, hormonal, cardiac and gastrointestinal medications, and steroid and thyroid hormones. A few patients reported that they had received other vaccinations (e.g., influenza or TBE) within the three-month window (Supplementary Data 3a and b).

### Genome-wide array data and analyses

Patients were genotyped with the Illumina Infinium OmniExpressExome-8 v1.6 array, and controls with the Illumina HumanOmniExpress 700 K array. Genotype calls were generated with the GenomeStudio software 2.0.3 (Illumina) using project-generated cluster files.

SWEDEGENE-COVID19-MYOPERIC genotype data and Twingene genotype data were quality controlled versus HRC1.1 Will Rayners qc script (v 4.2.9). The results were merged, and additional checks were performed.

Pre-imputation quality control (QC) included (i) gender checks, (ii) exclusion of variants with Hardy-Weinberg equilibrium p-value < 5×10^–8^ or minor allele frequency (MAF) < 0.5%, and (iii) exclusion of individuals with more than 2% missing variants.

Imputation was performed using the TOPMed reference panel^26–28^.

Post-imputation, the data was filtered on imputation quality (mimimac imputation quality R2 > 0.7) and converted to hard calls using PLINK v1.9.

The non-imputed data were merged with HapMap (release 23, 270 individuals,) and principal components (PCs) were calculated using PLINK v1.9. A graph of population stratification is shown as a scatter-plot with colors according to population in HapMap or swedegene cases/controls (Supplementary Fig. 4).

The GWAS analyses were performed using SAIGE with saddle point estimation with the setting that effect sizes for markers with p-values below 0.05 should be estimated using Firth’s bias-reduced logistic regression^29^. In short, SAIGE handles case-control imbalance and bias in results due to low-frequency variants. Odds ratio (OR) estimates for rare variants where low to no variation is seen among controls should be interpreted with caution i.e., we can detect a signal, but the estimate of effect is highly unreliable and can be affected by the Firth fallback of the analysis method^30^.

All analyses were adjusted for sex and the first four principal components. SNP effects were modeled as additive. The conventional genome-wide significant threshold *p* < 5 × 10^–8^ was used to correct for multiple testing. Results are presented as Manhattan plots. Results are presented on the Genome Reference Consortium human assembly GRCh38.

### Functional variants annotations

The top GWAS SNPs associated with myo-/pericarditis were intersected with different databases to obtain functional annotations, including:

(I) The candidate cis regulatory elements (cCREs) registry from the ENCODE project^31^, a cell and tissue-specific collection of representative DNase hypersensitivity sites (rDHSs) supported by either histone modifications (H3K4me3 and H3K27ac) or CTCF-binding data,
(II) HaploReg, a tool for exploring annotations of noncoding variants on haplotype blocks^32^.
(III) RegulomeDB, a database that provides functional context to variants or genomic regions of interest^33^.
(IV) The FORGE tool, which performs Functional element Overlap analysis of the Results of Genome Wide Association Study (GWAS) Experiments^34^.
(V) The JASPAR CORE 2022 collection of genome-wide predicted binding sites for transcription factors binding profiles^35^.
(VI) Gene expression levels from the Genotype-Tissue Expression (GTEx) project^15^ to investigate the tissue specific expression of the associated genes.

## Supplementary information

Supplementary Information

Supplementary Dataset 1-8

## Data Availability

Data from this project can be shared in a strictly anonymous form with other study groups, both national and international, to increase power. Internationally, this means countries within EU/EES, but also other countries outside the EU that, according to the European Commission, offer an adequate level of data protection (https://ec.europa.eu/info/law/law-topic/data-protection/international-dimension-data-protection/adequacy-decisions_en). Study and sample data will be made compatible with formats specified by international repositories to facilitate submissions. We will provide data at request in compliance with FAIR data management, if possible, according to GDPR.
