# Supplementary material for: Genome-wide association study of myocarditis and pericarditis following COVID-19 vaccination
Source: NPJ Vaccines. 2025 May 8;10:88. doi: 10.1038/s41541-025-01139-4 (PMC12062381; doi:10.1038/s41541-025-01139-4)
Supplement: Supplementary file 1 — Supplementary Information [file 41541_2025_1139_MOESM1_ESM.pdf]

Supplementary Information

Supplementary Figure 1

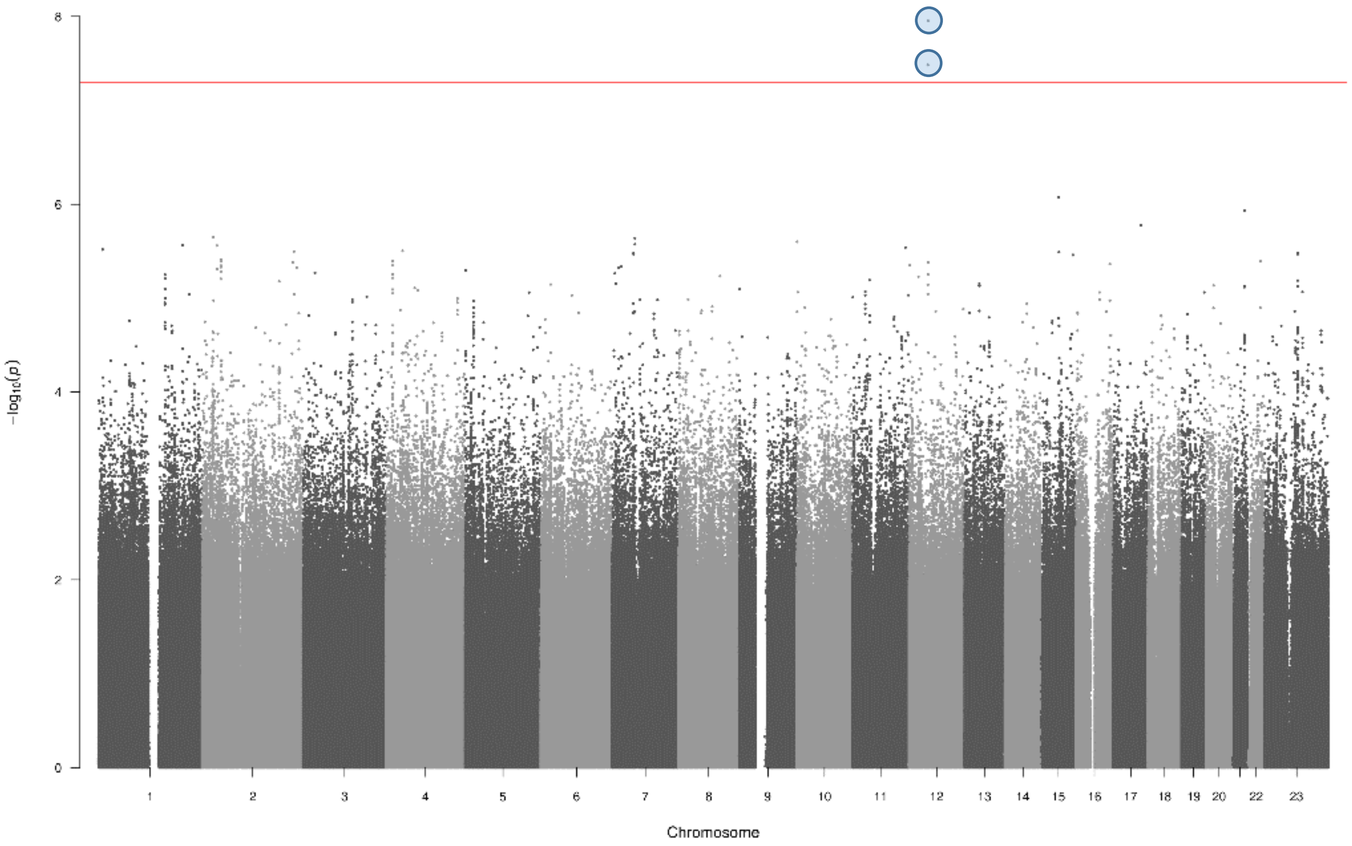

Supplementary Figure 1. SAIGE GWAS for all cases and all vaccines. Manhattan plot for the outcome all cases and vaccines compared with 4891 population controls. The red line denotes the significant level $p < 5x10^{-8}$.

Supplementary Figure 2

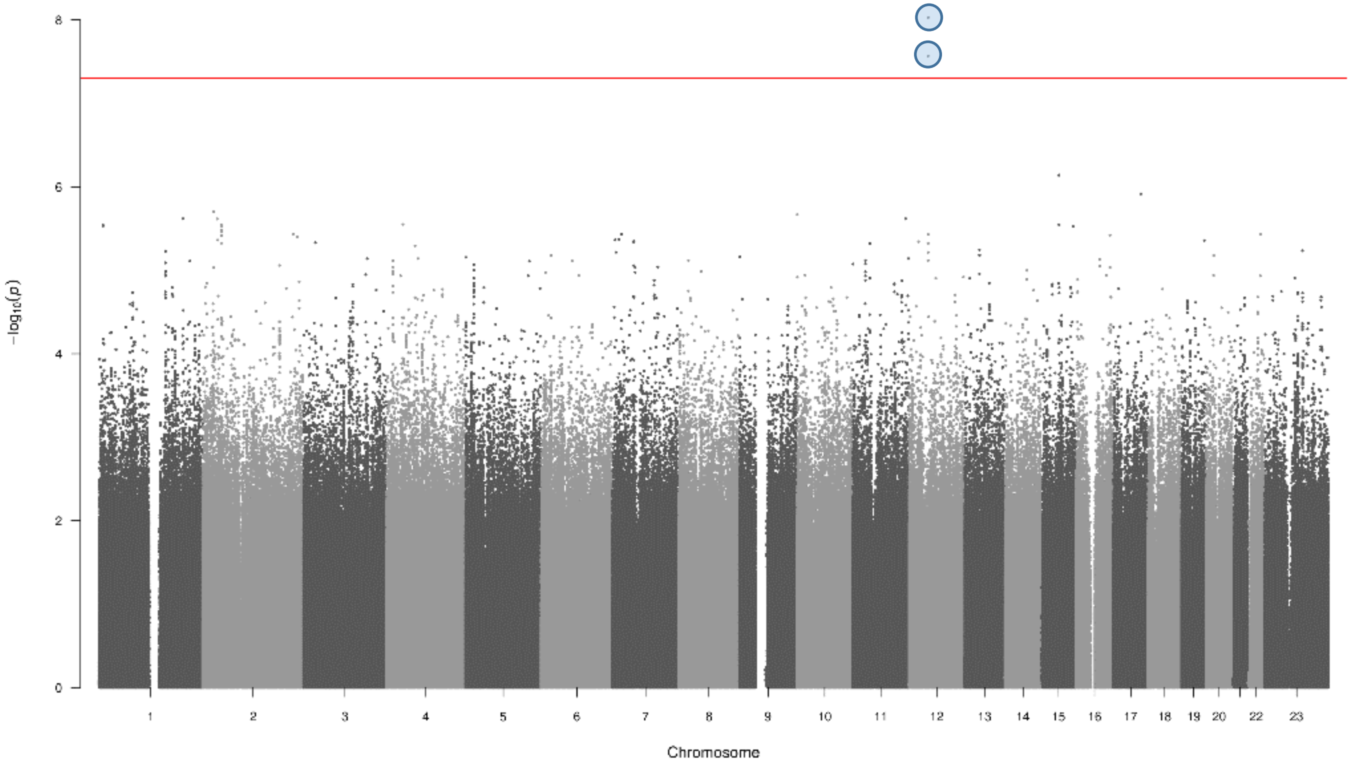

Supplementary Figure 2. SAIGE GWAS for all cases and Spikevax+Comirnaty. Manhattan plot for the outcome all cases after Spikevax or Comirnaty vaccination compared with 4891 population controls. The red line denotes the significant level $p < 5x10^{-8}$.

Supplementary Figure 3

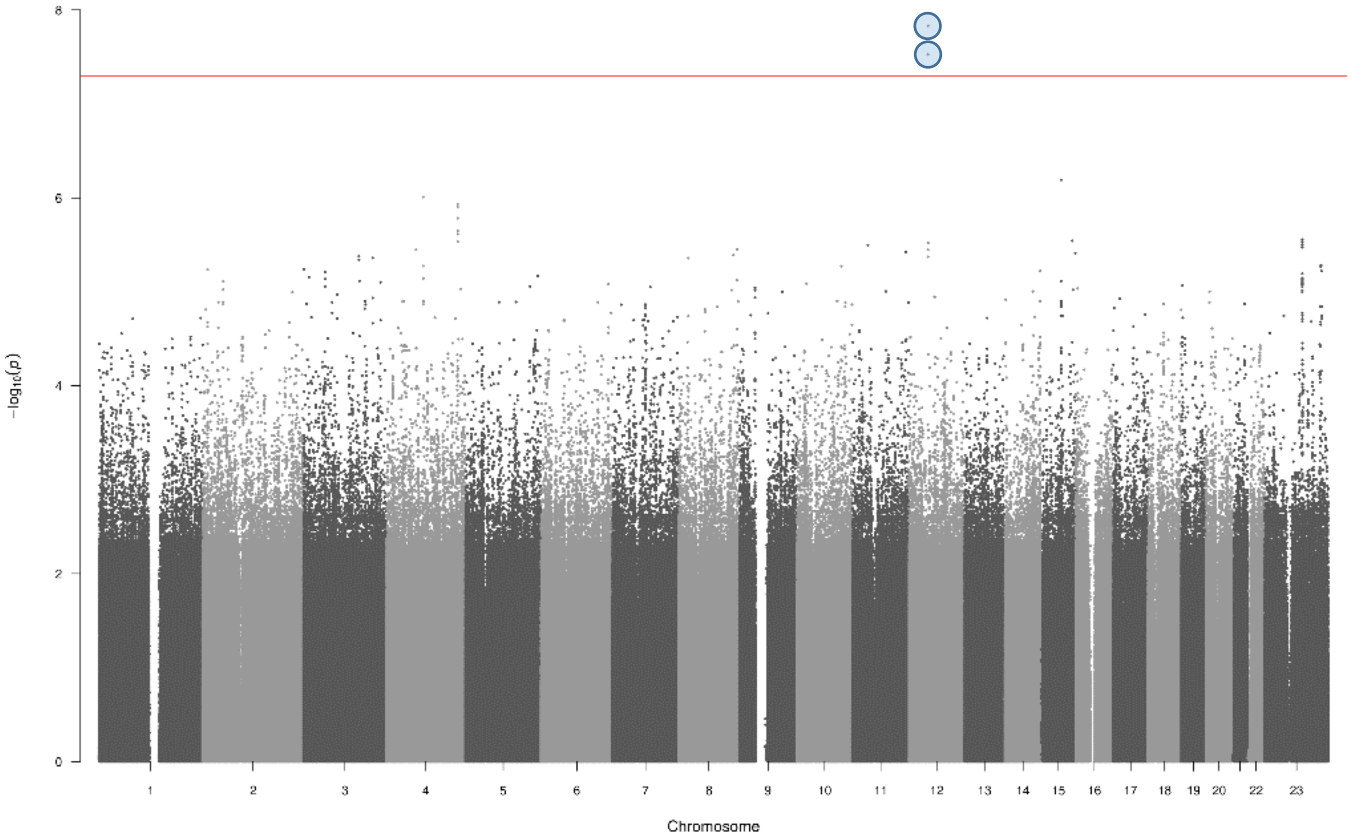

Supplementary Figure 3. SAIGE GWAS for cases: Pericarditis (including perimyocarditis) and Spikevax+Comirnaty. Manhattan plot for the outcome Pericarditis after Spikevax or Comirnaty vaccination compared with 4891 population controls. The red line denotes the significant level $p < 5x10^{-8}$.

Supplementary Figure 4

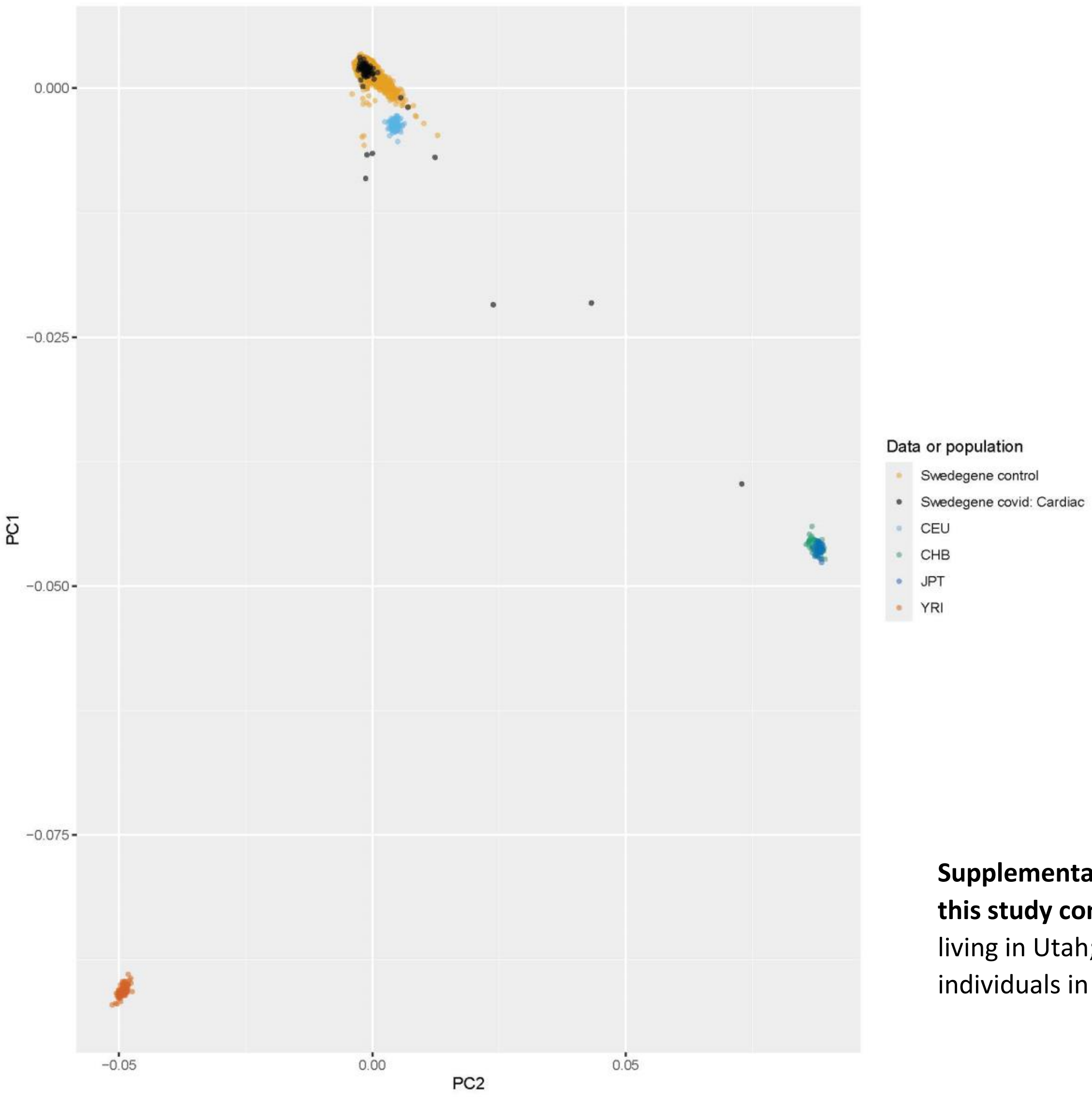

Supplementary Figure 4. PCAs of myo-/pericarditis cases and controls used in this study compared to four populations in HapMap. (CEU) European ancestry living in Utah; (CHB) Han Chinese individuals in Beijing, China; (JPT) Japanese individuals in Tokyo, Japan; (YRI) Yoruba in Ibadan, Nigeria.

Supplementary Data

Supplementary Data 1. Diagnostic elements reported in medical records from the different hospitals that treated the myocarditis, pericarditis and myopericarditis cases

Supplementary Data 2. (a) History of diseases before the onset. (b) Diseases classification.

Supplementary Data 3. (a) Medications in the 3 months before COVID-19 vaccination. (b) Medications groups

Supplementary Data 4. Top 60 results for the SAIGE GWAS all cases and all vaccines.

Supplementary Data 5. Top 60 results for the SAIGE GWAS for all cases and Spikevax+Comirnaty.

Supplementary Data 6. Top 60 results for the SAIGE GWAS for cases: Pericarditis (including perimyocarditis) and all vaccines.

Supplementary Data 7. Top 60 results for the SAIGE GWAS for cases: Pericarditis (including perimyocarditis) and Spikevax+Comirnaty.

Supplementary Data 8. Top 60 results for the SAIGE GWAS for cases: Myocarditis (including perimyocarditis) and Spikevax.
